# Multifocal intradural extramedullary myxopapillary ependymoma of the spine

**DOI:** 10.1007/s00432-026-06656-w

**Published:** 2026-11-09

**Authors:** Oday Atallah, Majd Oweidat, Sarah Teuber-Hanselmann, Kirollos Awad, Lambert Schless

**Affiliations:** 1 Department of Neurosurgery, Nordwest Hospital Sanderbusch, Teaching Hospital of Carl Von Ossietzky University Oldenburg, Am Gut Sanderbusch 1, 26452 Oldenburg, Germany; 2 College of Medicine, Hebron University, Hebron, West Bank Palestine; 3 Department of Neuropathology, Klinikum Bremen-Mitte, Bremen, Germany

**Keywords:** Myxopapillary ependymoma, Intradural extramedullary, Spine tumor, Multifocal tumor

## Abstract

**Background:**

Myxopapillary ependymoma (MPE) is an uncommon spinal ependymal tumor that usually arises as a solitary intradural extramedullary (IDEM) lesion in the conus medullaris, cauda equina, or filum terminale. MPE is designated WHO grade 2 since the 2021. Synchronous multifocal spinal MPE at initial presentation is rare and creates uncertainty regarding the extent and timing of surgery.

**Case presentation:**

A 34-year-old man with no significant medical history presented with sudden severe non-radicular low-back pain and no neurological deficit. Contrast-enhanced MRI of the brain and entire spine showed multiple enhancing IDEM lesions confined to the lumbosacral region (T12–L1, L2, L3–L4, L4–L5, and S1). The dominant right-sided T12–L1 lesion markedly compressed the conus medullaris/proximal cauda equina. Complete microsurgical resection of this lesion was performed, and histopathology confirmed MPE, WHO grade 2 (Ki-67 ≤ 2–3%). The patient declined radiation-oncology referral, and the remaining lesions were monitored with MRI and CSF cytology, which remained negative. Nine months later, he developed paresthesias and recurrent low-back pain, with enlargement of the L3–L4 and L4–L5 lesions. Both were completely resected in a second operation, while the stable L2 and S1 lesions were left in place. No new neurological deficit occurred after either operation. The patient subsequently underwent radiation-oncology evaluation and received radiotherapy, and the residual lesions were unchanged at 3 months.

**Conclusion:**

Multifocal spinal MPE may require individualized, staged management. Prioritizing symptomatic or compressive lesions can achieve decompression and tissue diagnosis while limiting multilevel surgical morbidity. Residual disease warrants long-term imaging surveillance, CSF assessment, and evaluation for radiotherapy.

## Introduction

Myxopapillary ependymoma (MPE) typically arises near the conus medullaris, cauda equina, or filum terminale, representing approximately 15–27% of all spinal ependymomas, and may present with low-back pain before neurological deficits become apparent (Montero et al. 2021; Dougherty et al. 2023; Qian et al. 2026). Although historically categorized as WHO Grade 1, MPE was reclassified as WHO Grade 2 in the 2021 World Health Organization Classification of Tumors of the Central Nervous System due to recurrence rates comparable to conventional spinal ependymomas (Louis et al. 2021; Kresbach et al. 2022).

Most MPEs present as solitary, slow-growing intradural extramedullary (IDEM) lesions; however, synchronous multifocal presentation at initial diagnosis without prior surgical intervention or documented cerebrospinal fluid (CSF) dissemination is rare (Qian et al. 2026; Tabor et al. 2022). In a surgical cohort of 101 patients, 13% had multifocal disease, and complete resection was substantially less frequent in that subgroup (Montero et al. 2021).

Managing multifocal MPE presents significant clinical and surgical challenges. While gross total resection (GTR) with tumor capsule preservation remains the cornerstone of treatment for solitary MPE (Qian et al. 2026; Nabors et al. 2020; Agrawal et al. 2025), attempting multi-level resection for widespread synchronous lesions can confer substantial operative morbidity, excessive blood loss, and spinal instability without immediate clinical benefit. Published adult reports describe varied approaches, including staged resection, observation of additional lesions, and radiotherapy, but provide limited guidance on which lesion to treat first or when to treat the remaining disease (Qian et al. 2026; Tabor et al. 2022; Almatrafi et al. 2023).

We present a neurologically intact 34-year-old man with multiple IDEM spinal MPE lesions, initially treated by resection of a compressive T12–L1 mass, followed by resection of previously recognized lumbar lesions after interval symptomatic and radiological progression.

## Case presentation

A 34-year-old man with no significant medical history presented to the emergency department with sudden-onset severe low-back pain. The pain did not radiate to either lower extremity. He reported no preceding trauma or constitutional symptoms. Neurological examination revealed no motor or sensory deficits, and bladder and bowel functions were preserved.

**Fig. 1:**
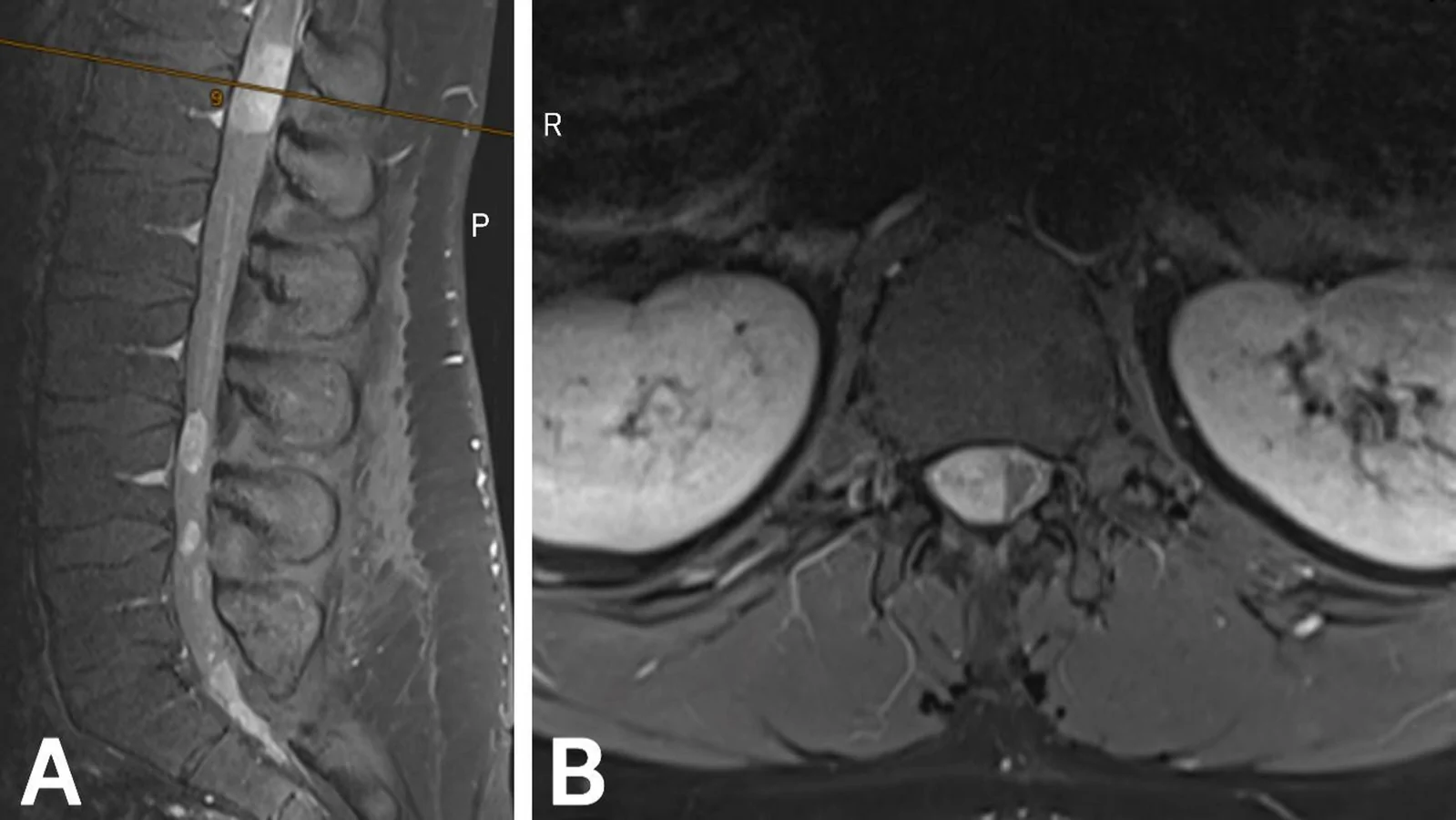
Initial MRI findings. Sagittal (**A**) and axial (**B**) contrast-enhanced T1-weighted MRI show multifocal IDEM enhancing lesions at T12–L1, L2, L3–L4, L4–L5, and S1. The dominant right-sided lesion at T12–L1 causes substantial compression and displacement of the conus medullaris/proximal cauda equina

Magnetic resonance imaging (MRI) of the brain and entire spine, performed before and after contrast administration, showed multiple well-defined enhancing IDEM lesions throughout the lumbar region. The dominant lesion was predominantly right-sided at T12–L1 and caused severe compression and displacement of the conus medullaris/proximal cauda equina (Fig. 1). No additional lesions identified.

Because of the marked mass effect produced by the dominant lesion, the multidisciplinary team recommended surgical treatment. The initial operation was intentionally limited to the T12–L1 lesion. The smaller lesions were asymptomatic, and resecting multiple levels during the same procedure was considered to confer substantial additional risk without immediate clinical benefit. Tissue diagnosis was also required to establish the tumor type and guide subsequent management.

Complete microsurgical resection of the T12–L1 lesion was performed through a right-sided hemilaminectomy with continuous monitoring of motor-evoked and somatosensory-evoked potentials (MEPs and SEPs). After opening the dura and arachnoid, a soft, grayish-white tumor was identified and carefully separated from the spinal cord using bipolar forceps. Neuromonitoring signals remained stable throughout the operation. No radiological or intraoperative evidence of intratumoral hemorrhage was identified. The cause of the sudden onset of pain remained uncertain. Postoperatively, the low-back pain resolved completely, no new neurological deficit occurred, and the wound healed by primary intention without complications.

Following the first operation, the patient was informed about the option of referral for radiation-oncology consultation regarding adjuvant radiotherapy for the residual multifocal disease. The patient declined the consultation. With resolution of the presenting pain, preserved neurological function, and asymptomatic residual lesions, close surveillance was pursued with serial contrast-enhanced MRI and CSF cytological assessment. Additional multilevel resection was considered to increase operative risk. CSF cytology remained negative for tumor cells during follow-up.

**Fig. 2:**
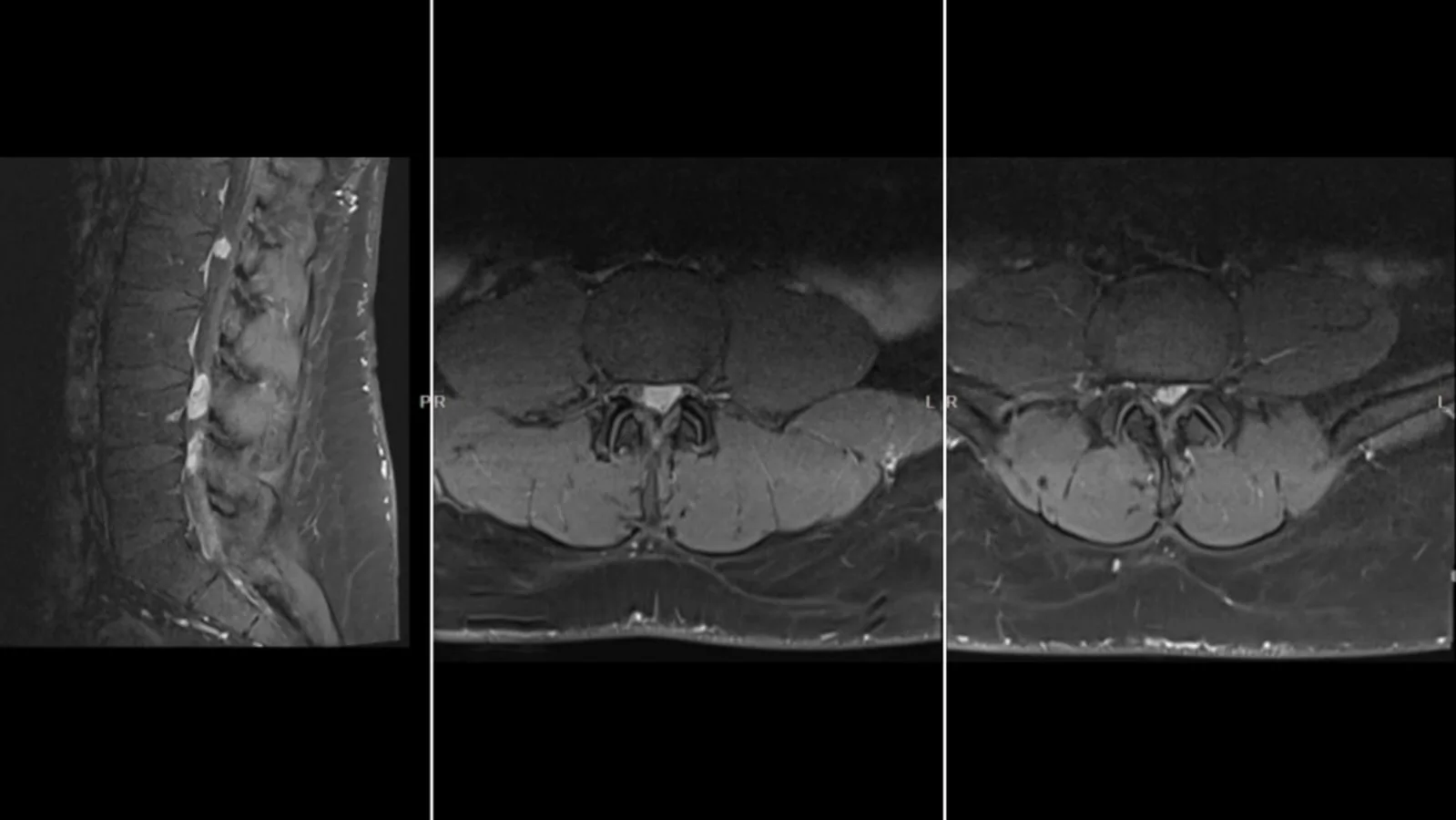
Interval progression after first-stage surgery. Contrast-enhanced MRI confirms complete resection of the T12–L1 lesion and shows interval enlargement of the predominantly left-sided IDEM lesions at L3–L4 and L4–L5. Smaller lesions at L2 and S1 remain stable

Nine months after the first operation, the patient developed intermittent reduced sensation in digits II–IV of the right foot and paresthesias involving both thighs. These symptoms progressively worsened over the following several months and were accompanied by recurrent intermittent non-radicular low-back pain. Contrast-enhanced MRI confirmed complete resection of the T12–L1 lesion but showed progression of the previously identified lesions at L3–L4 and L4–L5, which were predominantly left-sided, while smaller lesions at L2 and S1 remained stable (Fig. 2). At elective readmission, the patient was neurologically stable, with no definite objective motor or sensory deficits; the only abnormal finding was a mildly hyperactive left patellar tendon reflex.

Given the radiological progression and corresponding symptoms, a second operation was undertaken. During the surgery, the L3–L4 and L4–L5 interlaminar windows were exposed, followed by a left L4 hemilaminectomy. After dural opening, grayish-white tumor masses were found densely adherent to the nerve fibers. The lesions were sequentially and completely dissected from the neural tissue using microsurgical techniques, with preservation of the underlying nerve structures. During both procedures, the tumors were resected piecemeal, with intraoperative violation of the tumor capsules. The smaller L2 and S1 lesions remained stable and lay outside the exposure required for the progressive lesions. Their removal would have required additional exposure, increasing anticipated operative time, blood loss, and risk of spinal destabilization. Resection was therefore deferred because the anticipated additional morbidity outweighed the immediate benefit. This staged strategy addressed the symptomatic, progressive disease while preserving the option of adjuvant radiotherapy for the residual asymptomatic lesions. Postoperative MRI confirmed no residual tumor at the operated sites.

Following the second operation, the patient had no new neurological deficits, mobilized independently, and received regular physiotherapy. He was discharged on postoperative day 5 in good general condition, with outpatient follow-up planned. At 3 months, he remained neurologically stable, and MRI showed unchanged residual lesions at L2 and S1. He also underwent radiation-oncology evaluation and received radiotherapy.

**Fig. 3:**
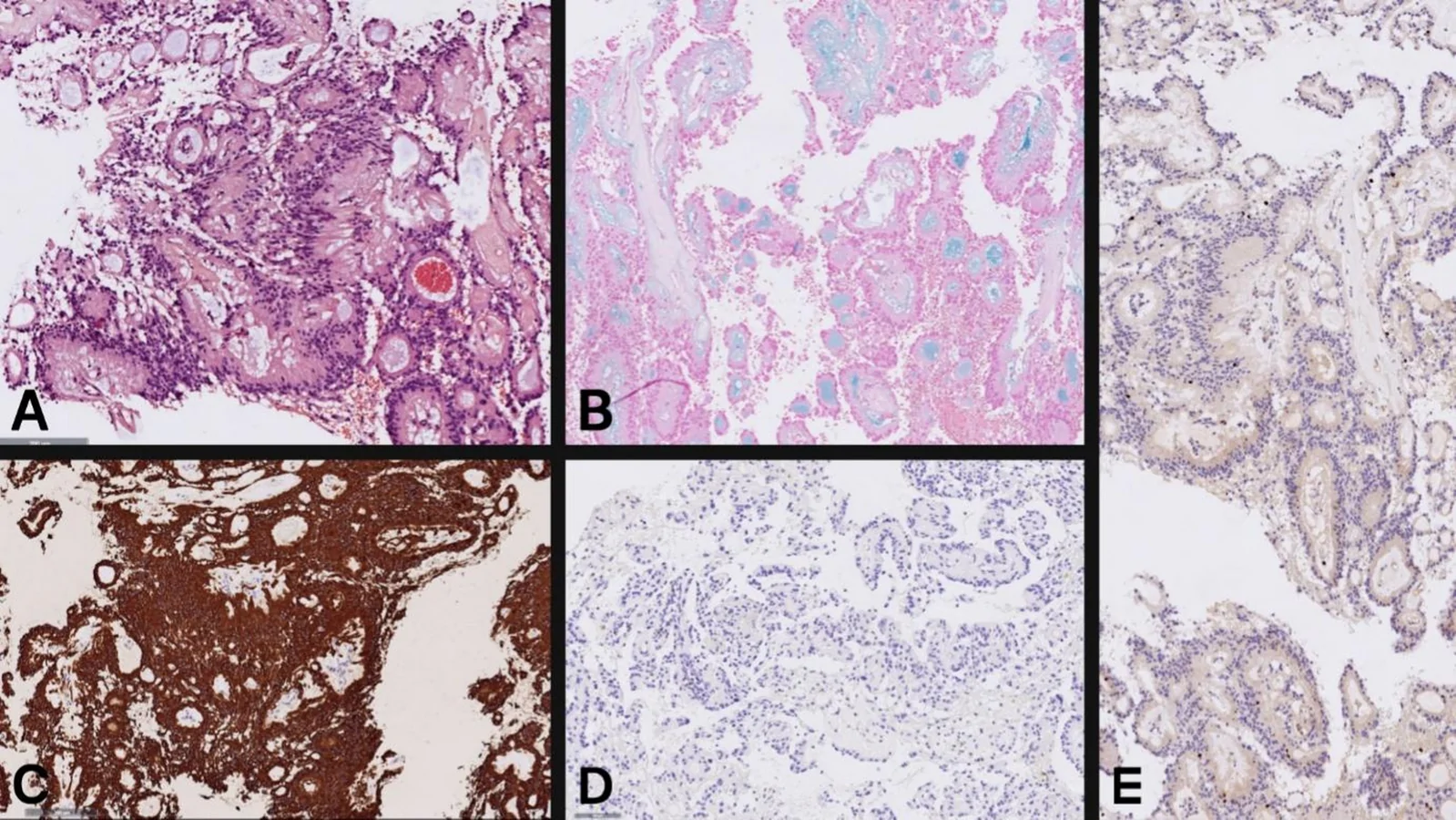
Histopathological features of the MPE. **A** H&E staining showing glial tumor cells arranged radially in papillary formations around hyalinized blood vessels and fibrovascular cores. **B** Alcian blue staining showing microcysts filled with myxoid material. **C** Strong, diffuse GFAP immunoreactivity in tumor-cell processes. **D** Negative nuclear staining for Olig-2. **E** Absence of dot-like perinuclear EMA staining. Findings were concordant in specimens from both operations

Histopathological findings were concordant in specimens from both operations (Fig. 3). The tumors contained glial cells arranged radially in papillary formations around hyalinized blood vessels and fibrovascular cores on hematoxylin-and-eosin staining (Fig. 3A). Microcysts contained Alcian blue-positive myxoid material (Fig. 3B). The cuboidal-to-elongated tumor cells had round-to-oval nuclei, inconspicuous nucleoli, and eosinophilic cytoplasm. Tumor-cell processes showed strong, diffuse immunoreactivity for glial fibrillary acidic protein (GFAP) (Fig. 3C), whereas the nuclei were negative for Olig-2 (Fig. 3D). Dot-like perinuclear epithelial membrane antigen (EMA) staining, typically observed in conventional ependymomas, was absent (Fig. 3E). The Ki-67 proliferation index did not exceed 2%–3% in either specimen. The histomorphology and immunohistochemistry findings established the diagnosis of MPE, World Health Organization grade 2, without histological evidence of malignancy. No further molecular testing was performed.

## Discussion

The marked T12–L1 compression justified treatment even though examination showed no neurological deficit. MRI defined the distribution and mass effect, but the pathological diagnosis rested on two concordant resections. Radially arranged glial cells around hyalinized vascular cores, Alcian blue-positive myxoid material, and GFAP immunoreactivity support MPE; negative Olig-2 and absent dot-like EMA staining are compatible with this diagnosis (Qian et al. 2026; Kresbach et al. 2022). A Ki-67 index of 2–3% describes low proliferative activity in the sampled tissue; it does not predict that other lesions will remain stable.

Multifocal IDEM MPE at initial presentation represents an exceptional entity in neuro-oncology, with only a few published cases in the medical literature (Qian et al. 2026; Tabor et al. 2022). Literature reviews show a male predominance (~ 79%) and a mean age of presentation around 35-years, with tumors preferentially clustering in the lumbosacral spine (Qian et al. 2026; Tabor et al. 2022). Although in this case, complete neuraxis imaging revealed no additional lesions and CSF cytology was negative, CSF-mediated dissemination among the identified spinal lesions cannot be excluded (Qian et al. 2026; Tabor et al. 2022). Therefore, “synchronous multifocal” describes their presence at initial diagnosis; whether they arose independently or through dissemination remains uncertain. This unresolved mechanism represents a limitation of the case.

Comparable reports show different courses. *Tabor* and colleagues resected a compressive lumbar lesion and then a sacral lesion in two operations, leaving tumor adherent to S2 roots and administering focal radiotherapy (Tabor et al. 2022). *Almatrafi* and colleagues treated the dominant T12–L3 mass in a 28-year-old man with additional lumbar lesions (Almatrafi et al. 2023). In a 21-year-old man with lesions at D12–L3, L4–L5, and S1–S2, Agrawal and colleagues reported a new thoracic lesion six months after resection (Agrawal et al. 2025). Qian and colleagues described four patients with multifocal MPE; one developed relapse during follow-up (Qian et al. 2026). In contrast, Abdu and colleagues reported new spinal nodules after resection of an initially solitary L2 tumor, a different sequence from disease already visible on this patient’s first scan (Abdu et al. 2021).

The staged approach here prioritized decompression and tissue diagnosis, then addressed enlarging, symptomatic lesions while limiting exposure of additional neural tissue. Its rationale is consistent with the difficulty of resecting disease across multiple levels. In Montero and colleagues’ 101-patient cohort, gross-total resection was achieved in 15% of multifocal versus 84% of solitary cases; incomplete resection and sacral involvement were associated with recurrence (Montero et al. 2021; Kresbach et al. 2022). In Dougherty and colleagues’ smaller cohort, seven of nine patients with subtotal resection required another operation, compared with none of 19 patients after gross-total resection (Dougherty et al. 2023). These observational findings are not an individual risk estimate.

The primary dilemma in multifocal MPE centers on balancing maximal surgical cytoreduction against procedure-related morbidity. In our patient, a single-stage total resection of all lesions would have required extensive multi-level laminectomies, significantly increasing the risk of spinal instability, hemorrhage, and neurological deficit. Therefore, a staged, symptom-guided microsurgical strategy was implemented. Long-term outcomes for multifocal MPE remain guarded, as residual un-resected lesions harbor an inherent risk for delayed progression or leptomeningeal seeding (Qian et al. 2026; Nabors et al. 2020; Agrawal et al. 2025).

Preservation of capsule integrity is an important surgical goal in MPE, as intraoperative capsule violation has been associated with increased local recurrence risk, due to microscopic seeding (Nabors et al. 2020; Agrawal et al. 2025). In our patient, capsule disruption necessarily occurred during both piecemeal resections.

Staged surgery directed by symptoms and radiological progression may represent an individualized management option in selected patients with multifocal spinal MPE, particularly when extensive multilevel resection would carry substantial morbidity. Residual lesions require continued imaging surveillance and evaluation for radiotherapy. This report is limited by its single-case design and the short clinical and radiological follow-up of 3 months after the second operation. Although the residual lesions remained stable at that assessment, this interval is insufficient to evaluate long-term tumor control or the durability of the staged treatment strategy.

## Data Availability

All data generated or analyzed during this study are included in this published article.
